# Performance Improvement for the CuCrZr Alloy Produced by Laser Powder Bed Fusion Using the Remelting Process

**DOI:** 10.3390/ma17030624

**Published:** 2024-01-27

**Authors:** Lianyong Xu, Yaqing Zhang, Lei Zhao, Wenjing Ren, Yongdian Han

**Affiliations:** 1 School of Materials Science and Engineering, Tianjin University, Tianjin 300350, China; 2 Tianjin Key Laboratory of Advanced Joining Technology, Tianjin 300350, China

**Keywords:** additive manufacturing, laser powder bed fusion, remelting process, CuCrZr alloys

## Abstract

Owing to the high optical reflectivity of copper powder, the high-performance fabrication of copper alloys in the laser additive manufacturing (AM) field is problematic. To tackle this issue, this study employs the remelting process during laser powder bed fusion AM to fabricate defect-free and high-performance CuCrZr alloy. Compared to the non-remelting process, the remelting process yields finer grains, smaller precipitates, denser dislocations, and smaller dislocation cells. It realizes not only the dense molding of high laser reflectivity powders but also excellent mechanical properties and electrical conductivity (with an ultimate tensile strength of 329 MPa and conductivity of 96% IACS) without post-heat treatment. Furthermore, this study elucidates the influence of complex thermal gradients and multiple thermal cycles on the manufacturing process under the remelting process, as well as the internal mechanisms of microstructure evolution and performance improvement.

## 1. Introduction 

Copper and copper alloys have excellent mechanical and physical properties, as well as high electrical and thermal conductivities. They are widely utilized in electronics, manufacturing, national security, aerospace, and other industries and significantly influence both the economy and technological advancements. Currently, the Cu–Ni–Si, Cu–Fe–P, and Cu–Cr series are the most popular copper alloys with excellent properties [1,2,3,4,5]. Among them, the CuCrZr alloy is prominent because of its superior conductivity and high strength in the power electronics industry. Most previous studies on CuCrZr alloys have employed traditional manufacturing techniques, but they frequently merely provide simple standard shapes, which is challenging for producing complex structures. With the advancement of additive manufacturing (AM) technology, numerous studies have demonstrated AM’s machinability, including the direct formation of nearly fully dense specimens with good mechanical characteristics [6,7,8]. Laser powder bed fusion (PBF-LB/M) is a typical powder bed AM technology, also known as selective laser melting. In PBF-LB/M, a high-energy laser beam is used to scan the metal powder bed layer-by-layer, following the three-dimensional path planned in a computer-aided design slice model. Then, the scanned metal powders melt and solidify to realize metallurgical bonding and finally, accumulating to form a three-dimensional solid. PBF-LB/M overcomes the challenges posed by conventional methods in producing metal parts with intricate geometries. Some alloys produced through AM techniques, including high-entropy alloys, Cu-based shape memory alloys, titanium alloys, and stainless steels, are currently employed in practical engineering fields such as aerospace and medical devices. Studies have also focused on the process, heat treatment, microstructure, properties, and defects such as cracks in laser AM [9,10,11,12,13,14].

Recently, with the maturity of AM technology, the development of PBF-LB/M technology, and the rising demand for complex functional copper alloy samples, research has shown that PBF-LB/M technology can yield complex and accurate copper alloy samples. Hazeli et al. [15] studied the effects of microstructure and topology on the quasi-static and dynamic behaviors of the lattice structure of copper alloys. Wallis et al. [16] produced different dense components with micrometer-scaled structures, such as a double-deck microchannel device, a lattice structure for filter systems, and the housing for electronic devices with integrated cooling structures. Zhao et al. [17] investigated the effect of melt pool overlapping on microstructure and properties during the PBF-LB/M process, including the effects of overlap on defect and microstructure evolution. They offer a new way to improve the microstructure and mechanical properties of materials by optimizing overlap conditions. Wegener et al. [11] fabricated CuCrZr parts using PBF-LB/M technology and investigated the relationship between different heat treatment conditions and maximum hardness and maximum conductivity.

However, all previously produced copper alloy powders exhibit high optical reflectivity (especially for 1070 nm in laser wavelength). Owing to the low laser absorption and high thermal conductivity of the CuCrZr alloy, fabricating specimens with high relative density (DR) by PBF-LB/M is difficult. Many modification techniques have been studied to resolve these drawbacks. Jadhav et al. [18] proposed a surface modification method for the powder and doubled the optical absorption of the alloy produced by the PBF-LB/M process. The powder was modified by the outward diffusion of chromium in a nitrogen atmosphere, which formed a rim around the powder particles. Zhang et al. [19] prepared a high-strength and highly conductive alloy using a high-power laser during the PBF-LB/M process and compared the microstructure, mechanical properties, and conductivity before and after heat treatment. Tidén et al. [20] investigated laser bed fusion of Cu using graphene oxide-coated powder, which exhibited reduced reflectivity, improved printability, and increased densities using a laser power of 500 W. Tang et al. [21] used a short-wavelength laser (515 nm) during the PBF-LB/M process, optimized the process parameters, and analyzed the mechanical and electrical properties based on improving the laser absorption rate of the powder. The efficiency of the remelting process between the layers and the absorption of laser energy increased with decreasing layer thickness and powder size. Qu et al. [22] employed small-sized powder and thin layer thickness to inhibit the growth of columnar grains and enhance the mechanical properties via high-precision PBF-LB/M.

The aforementioned methods can realize the PBF-LB/M manufacturing of copper alloys, but they simultaneously increase PBF-LB/M production costs due to powder treatment or laser upgrades. Numerous studies have been conducted on optimizing processes under current conditions, one of which is the remelting process. Nowadays, the most commonly used laser power is less than 500 W. To produce dense specimens, the non-remelting process usually requires high-energy densities. Comparatively, the remelting process requires lower energy densities, but it takes longer to finish. However, for producing high-density specimens, a long manufacturing time is worth considering. To produce defect-free copper alloy samples using normal equipment, developing and popularizing the processing of copper alloy samples through the PBF-LB/M process is beneficial.

Liu et al. [23] studied the effect of the remelting process on the forming quality of PBF-LB/M-processed cemented carbide and found that remelting can effectively improve the surface quality and relative density of specimens. Chou et al. [24] studied the effects of different laser energy densities and remelting on the uniformity of deposition trajectories by melting a layer of aluminum powder on a Cu substrate, and they found that remelting is an effective strategy for improving homogeneity. Currently, few studies have been conducted on the necessity of the PBF-LB/M remelting process and the relationship between pre-melting and remelting of the CuCrZr alloy. Moreover, systematic research on the internal mechanisms of performance improvement stemming from the remelting process for the CuCrZr alloy is lacking.

In this study, a remelting process with a low-power (<400 W) infrared laser was employed to produce high-quality and defect-free CuCrZr alloys through PBF-LB/M. A lap model of the molten pool (MPL model) was used to estimate the process window and design the optimum processing parameters. A comparison of the microstructure, mechanical characteristics, and conductivity of specimens manufactured using the remelting and non-remelting processes showed that the remelting process produces smaller grains and precipitates and improves the overall performance. These characteristics enable CuCrZr alloy components to be employed in thermally conductive devices in the power sector and in engine blades at high temperatures in the aerospace industry. Such a remelting process can also be readily adapted for the AM of other reflective metals for various novel and potential applications.

## 2. Materials and Methods

### 2.1. Materials and PBF-LB/M Fabrication

**Figure 1:**
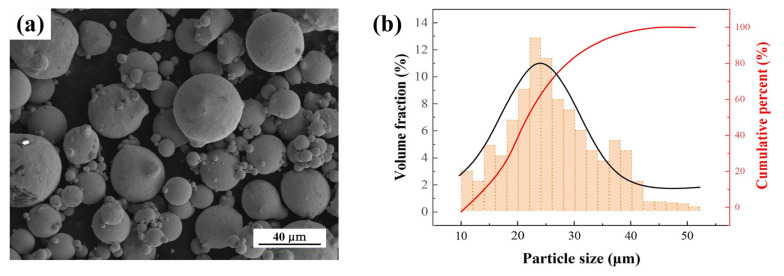
Characterization of raw powders: (**a**) SEM images of CuCrZr powders and (**b**) particle size distribution.

The powder utilized in the experiment was gas-atomized Cu–0.82 mass% Cr–0.48 mass% Zr powder (Willari Co., Ltd., Xuzhou, China). Figure 1 presents the scanning electron microscope (SEM) images and particle size distribution of the virgin powders, with a particle size distribution between 15 and 53 μm and a D_90_ of 52.7 μm.

A Renishaw AM400 SLM system (Renishaw, London, UK), equipped with a 400 W laser optical system and a wavelength of 1064 nm, was used to fabricate CuCrZr specimens. Stainless steel 316 L was used as the substrate material. The manufacturing process was completed in a high-purity argon atmosphere to avoid oxidation, and the oxygen level in the working chamber was maintained below 100 ppm.

The PBF-LB/M process employed a laser scanning strategy that rotated 67° from layer to layer during the non-remelting and remelting processes to prevent energy and stress accumulation. The scan path for each layer of the remelting process is the same as the path of the non-remelting process. The substrate was preheated to 170 °C before building the specimens to improve the wettability of the molten pool and the energy absorption of the powders.

### 2.2. Process Optimization

A three-factor crossover experiment with a laser power of 300–400 W, exposure time of 120–200 μs, and hatch spacing of 50–90 μm was initially performed, including remelting and non-remelting processes for 5 × 5 × 5 mm^3^ cube-shaped specimens to establish the process window and obtain preliminary data. The layer thickness and laser point space were kept constant at 30 and 60 μm, respectively. The volumetric laser energy density was calculated using Equations (1) and (2):

(1) $$E=\frac{P}{v\times h\times t}\;$$

(2) $$v=\frac{d}{\theta }\;$$

where *E*, *P*, *v*, *h*, *t*, *d*, and *θ* are the volumetric laser energy density, laser power, laser scan speed, hatching space, layer thickness, laser point space, and exposure time, respectively. Table 1 presents the parameters selected for the process optimization window. Notably, the remelting process was realized by exposing the specimens to the laser twice under identical process parameters. Furthermore, to distinguish the energy density of the remelting process, it was denoted as *E^re^*.

The size of the melt pool in the topmost layer in the building direction was measured by an optical microscope after etching to investigate the lapping of the molten pool. The preliminary data, which served as the source data for the lap model, included the molten pool size and the relative density of the specimens under various parameter combinations, such as the width and depth of the molten pool.

**Figure 2:**
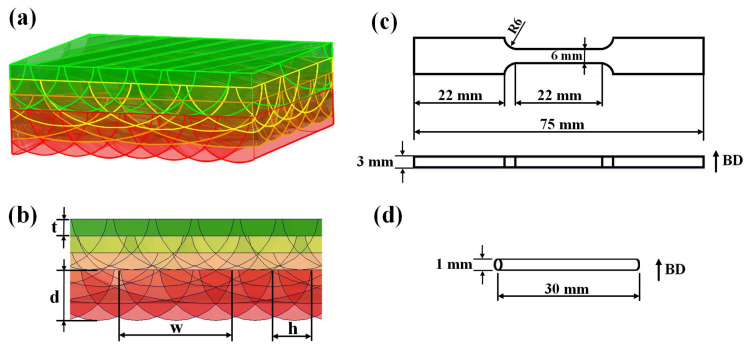
(**a**) Schematic of the MPL model, (**b**) cross-section of the orthogonal molten pool, (**c**) schematic diagram of the tensile specimen, and (**d**) conductivity testing specimen.

The MPL model was established based on the molten pool size, as shown in Figure 2. It simulates the lap connection of each molten pool channel in the PBF-LB/M process. The model reveals the relationship between the process parameters and defects. Hatching space, molten pool width (w), and depth (d) are the independent variables of the model. The lap rate of the molten pool (R_L_) denotes the number of times the molten pool overlaps during the manufacturing process. The R_L_ under various process parameters in the unified area extent can be determined by the area analysis function of Computer Aided X Alliance software. Based on the measurements, a series of models was established. The simulation results were used to optimize the best process parameters by analyzing the R_L_ and the microstructure under the same parameters. A symmetrical semi-elliptical shape was the default shape of the melt pool for the model, regardless of the residual height.

### 2.3. Microstructure and Properties Characterization

The densities of the bulk samples were measured according to Archimedes’ principle. The metallographic along the building direction and fracture morphologies of the samples were characterized using an optical microscope (OM, Axio Vert.A1, ZEISS, Aalen, Germany) and scanning electron microscope (SEM, Sigma-300, ZEISS, Aalen, Germany), equipped with an energy dispersive spectrometer (EDS) system. For the microstructure analysis, based on standard procedures, the specimens were etched with a mixed solution of 3 g of FeCl_3_·6H_2_O, 10 mL of HCl, and 30 mL of distilled H_2_O for 12 s after grounding and polishing. For the electron backscattered diffraction (EBSD) analysis, the specimens were automatically polished with an argon ion beam polisher (Fischione, Export, PA, USA) to yield high-quality cross sections. EBSD tests were conducted at a voltage of 20 kV and a step size of 1 μm, using a high-resolution detector (Bruker, e-Flash, Aalen, Germany) with post-processing software (Esprit 4.0, Max Plank, Hürth, Germany) to analyze the grain size, crystal orientation, and grain boundary deviation. Transmission electron microscopy (TEM, Tecnai G2 F30, FEI, Hillsboro, OR, USA) with energy dispersive X-ray spectroscopy (EDX) was used to analyze the microstructure and composition distribution of nanocrystals, as well as dislocations and precipitates, at an acceleration voltage of 200 kV. TEM specimens were prepared by twin-jet electro-polishing, using a Struers TenuPol-5 system inside a mixture solution of 4 vol% perchloric acid and 96 vol% ethanol at −30 °C, followed by ion-beam thinning using a Gatan 691 system. X-ray diffraction (XRD, D8 ADVANCE, Bruker, Karlsruhe, Germany) was performed at 40 kV and 40 mA and scanned in the range of 30°–100° with an interval of 0.02° for 0.1 s counting time to analyze the crystal structure and phase composition of the specimens.

The test specimens were horizontally built, and the building direction followed the thickness or diameter direction. The process parameters for the test specimens were chosen based on the process optimization results for both remelting and non-remelting. The original gauge length and parallel length in the tensile specimens were 22 mm and 75 mm. Tensile tests were conducted on a mechanical testing machine (LD26, Lishi, Shanghai, China) at room temperature by employing a constant strain rate of 2.5 × 10^−4^ s^−1^ and quasistatic loading. Each tensile test was performed at least thrice under the same conditions. Hardness tests were conducted on a HV-10CCD (CANY, Shanghai, China) microhardness tester at a load of 300 g for 15 s. Before performing the hardness tests, the surface of each specimen was polished, and 25 points were evenly chosen for testing. Conductivity tests were conducted at room temperature on a Tonghui TH2515 testing machine equipped with a source measure unit (Keithley 2460, Keithley, Cleveland, OH, USA). The source mode was set to DC voltage sweep with a test voltage range from −0.5 to 0.5 V in 0.02 V steps. The conductivity test specimen was a smooth-surfaced cylinder with a cross-sectional diameter of 1 mm and a length of 30 mm. In addition to electrical conductivity, thermal conductivity is also one of the important parameters of copper alloys. The thermal conductivity of CuCrZr alloys is calculated using the Wiedmann–Franz law [21,25,26]:

(3) λ=LσT

where λ is the thermal conductivity, *L* is the Lorentz number at 2.41 × 10^−8^ Ω·WK^−2^, *σ* is the resistivity (the reciprocal of conductivity), and *T* is the temperature.

## 3. Results

### 3.1. Parameter Optimization of the CuCrZr Alloy

**Figure 3:**
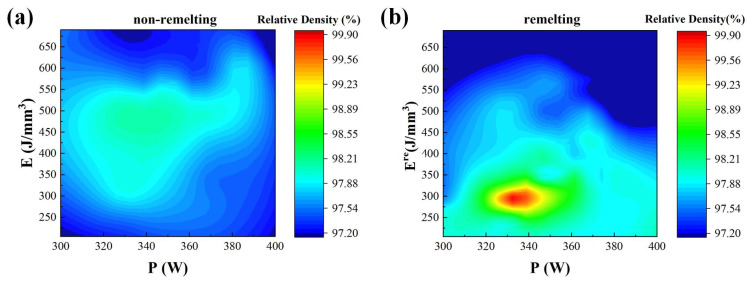
Parameter cloud plot of the relative densities of the PBF-LB/M specimens: (**a**) non-remelting process and (**b**) remelting process.

Figure 3 illustrates the cloud plot of the relative densities that are affected by various volumetric laser energy densities (taking laser power as an example). The highest relative density obtained by the remelting process reaches 99.8%. Figure 3b shows that the high density is concentrated in a particular region (the red area), and the optimum parameter can be found by matching the experimental parameters near the center of the red area. Furthermore, the optimal process window is more concentrated, signifying that the parameter combination under the remelting process is more likely to achieve high density. The density is significantly reduced when *E* is outside the range of 400–500 J/mm^3^ (non-remelting process) or *E^re^* is outside the range of 270–310 J/mm^3^ (remelting process). This is caused by defects, such as keyholes or lack of fusion (LOF), as shown in combination with the microstructure analysis. Similar findings were also obtained in the non-remelting processes. However, the remelting process clearly has a more focused and regular process window that allows for higher densities than the non-remelting process.

**Figure 4:**
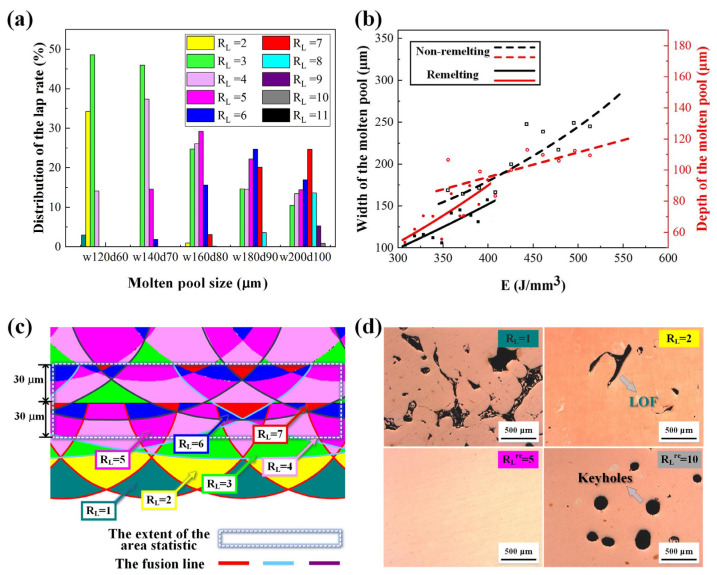
(**a**) R_L_ distribution based on the MPL model analysis (take *h* = 90 μm as an example), (**b**) fitting curve of the process parameters and molten pool sizes, (**c**) schematic of the statistical method of R_L_, and (**d**) OM images of the specimen surface corresponding to various R_L_.

To further compare the remelting and non-remelting processes and determine the optimal parameters, the lap rate distribution under various parameters was analyzed using the MPL model, as shown in Figure 4. Figure 4a,b presents the R_L_ distribution and the fitting curves of the process parameters and molten pool sizes. Figure 4c displays the schematic of the statistical method of R_L_. Figure 4d presents the OM images of the specimen surface corresponding to various R_L_, where R_L_ = 1 and R_L_ = 2 represent the non-remelting specimens, and R_L_ = 5 and R_L_ = 10 represent the remelting specimens. The best range of R_L_ was determined to be 4–6 for the non-remelting process and 3–7 for the remelting process by combining the statistical results of the R_L_ and density of the specimen in different parameters. Finally, the optimum parameter combination for the non-remelting (*P* = 330 W, *v* = 300 mm/s, *h* = 0.07 mm, and DR = 98.9%) and remelting (*P* = 330 W, *v* = 400 mm/s, *h* = 0.09 mm, and DR = 99.8%) processes was selected to fabricate specimens for analyzing the microstructure and exploring the mechanical and electrical properties.

### 3.2. Microstructure Characterization of the CuCrZr Alloy

**Figure 5:**
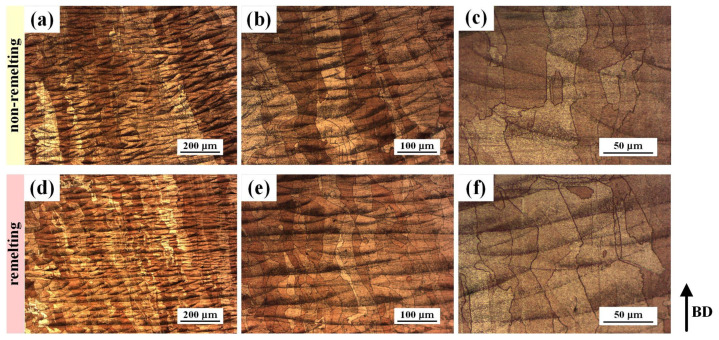
OM images of (**a**–**c**) the non-remelting process and (**d**–**f**) remelting process specimens.

**Figure 6:**
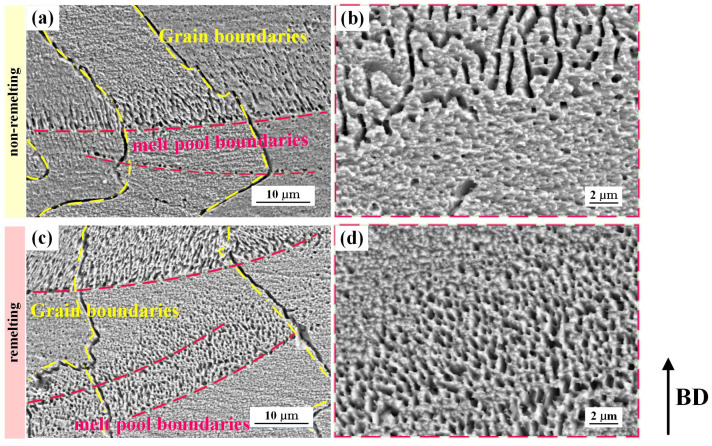
SEM images of (**a**,**b**) the non-remelting process, (**c**,**d**) remelting process specimens; (**b**,**d**) high-magnification SEM images around the melt pool boundary.

Figure 5 depicts the OM images of the PBF-LB/M CuCrZr specimens. The melt pool boundary is obviously visible after chemical etching. For specimens with density values up to 99%, microscopic defects are essentially invisible. Moreover, layers of overlapping molten pools and columnar crystals that stretch across multiple layers and expand in the manufacturing direction can clearly be observed, with a distinct inclination toward epitaxial growth. This phenomenon has also been reported in some related studies [19,27,28]. Similar findings were observed by the SEM, particularly, with substructures being observed. These substructures were concentrated near the bottom of the molten pool and extended perpendicular to the melt pool boundary toward the center of the molten pool, as shown in Figure 6. The substructure size of the remelting process was smaller and denser than that of the non-remelting process.

**Figure 7:**
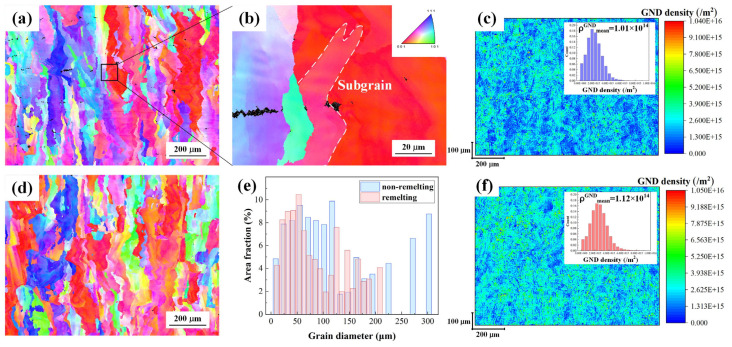
EBSD detection and analysis results: IPF for (**a**) non-remelting and (**d**) remelting process specimens along the BD, (**b**) high-magnification images of (**a**), GND for (**c**) the non-remelting and (**f**) remelting process specimens, and (**e**) cumulative frequency of different grain diameters for the specimens.

Figure 7a,d displays the inverse pole figures (IPFs) of two CuCrZr alloys along the building direction under the preferred non-remelting and remelting process parameters. Due to the resolution limitations, only the Cu phase with face-centered cubic (FCC) structure was detected. Precipitates could not be distinguished via EBSD. Under high magnifications, the sub-grains were differentiated from the grains. Due to the 67° rotation scanning strategy and the direction of heat dissipation during solidification, the grains vertically meandered in the optimal direction, that is, perpendicular to the normal direction of the maximum curvature point of the molten pool. The average grain size of the remelting specimen was 85.6 μm, which is smaller than that of the non-remelting specimen (126.1 μm). Figure 7c,f presents the geometrically necessary dislocations (GNDs). The GND densities for non-remelting and remelting processes were 1.01 × 10^14^ and 1.12 × 10^14^, respectively.

**Figure 8:**
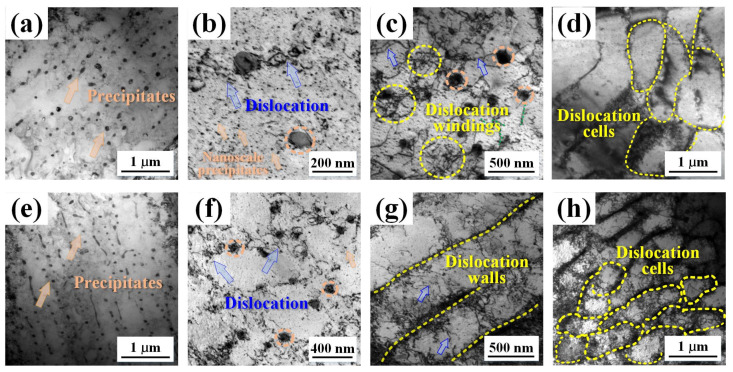
TEM image of the (**a**–**d**) non-remelting and (**e**–**h**) remelting process specimens: (**a**,**e**) the precipitates, (**b**,**f**) the dislocation, (**c**) the dislocation winding, (**g**) the dislocation walls, and (**d**,**h**) the dislocation cells.

The microstructure and phase composition of specimens were verified via TEM, as shown in Figure 8. Across the matrix in the bright-field image, several nanoprecipitates, dislocation tangles, and dislocation cells were strewn. The average size of the precipitates in the remelting process specimen was 77 nm, and in the non-remelting process specimen, it was 107 nm. The non-remelting specimen contains more nanoscale precipitates, as shown in Figure 8b. Furthermore, countless dislocation lines were observed encircling the precipitated phases, which created a consistent network and formed a cell-like substructure known as a subcrystalline grain or dislocation cell. The size of the dislocation cells in the remelting and non-remelting process specimens was 633 and 1200 nm, respectively. 

**Figure 9:**
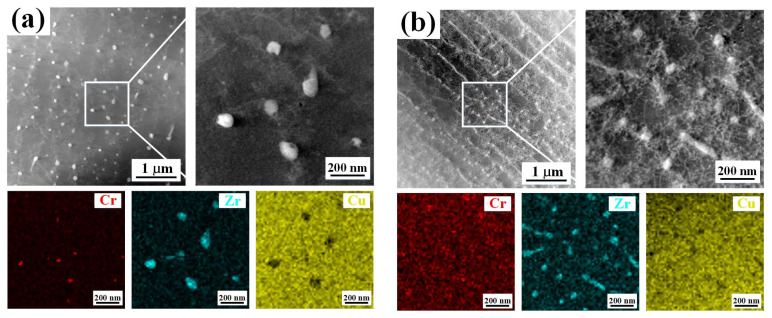
TEM mapping analysis of the (**a**) non-remelting and (**b**) remelting process specimens.

**Figure 10:**
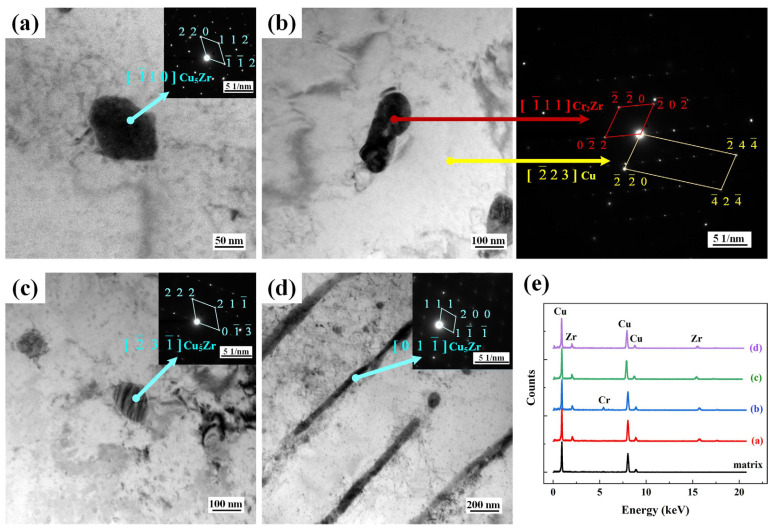
TEM images of precipitates in the matrix and SAED pattern: (**a**,**b**) non-remelting process, (**c**,**d**) remelting process, and (**e**) TEM-EDX for the matrix and the precipitates of (a–d).

Figure 9 displays the TEM-energy dispersive X-ray (TEM-EDX) elemental mapping results. The figure shows that the precipitates in the matrix are composed of Cr and Zr. Furthermore, a “core–shell structure” formed in the non-remelting process when the Cr precipitate was surrounded by the Zr precipitate like a nucleus. The TEM image verified the overlapping of two elements. This structure was not obvious in the remelting specimen. In the selected area electron diffraction (SAED) images, as shown in Figure 10, all the nanometer precipitates exhibited an FCC structure despite the different band shaft indices. The nanoprecipitate was analyzed via TEM-EDX to confirm its composition (Figure 10e). Cu_5_Zr complexes constituted the majority of the precipitates, containing $\left[\bar{1}10\right]$_Cu5Zr_, $\left[\bar{2}3\bar{1}\right]$_Cu5Zr_, and $\left[01\bar{1}\right]$_Cu5Zr_. Some of the precipitated phases in the non-remelting specimen contained Cr_2_Zr complexes. No CrZr complexes were observed in the precipitated phases in the remelting specimen.

**Figure 11:**
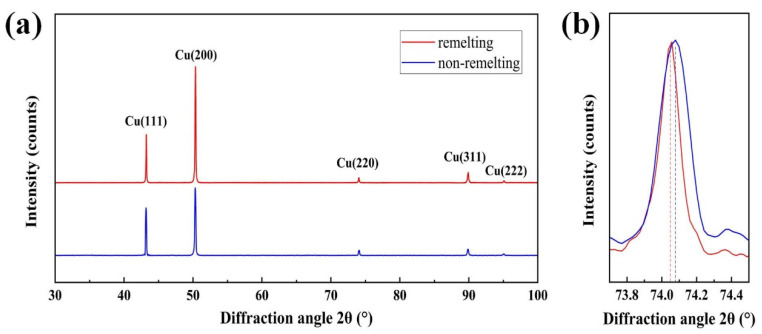
XRD pattern of (**a**) remelting and non-remelting specimens, (**b**) magnified views of spectra around 2θ = 74°.

Figure 11 depicts the XRD patterns of the two specimens from 2θ = 30°–100°. No diffraction peak of the other phase was observed due to low amounts of the two elements in the alloys. Both XRD patterns exhibited diffraction peaks corresponding to the (111), (200), (220), (311), and (222) crystal planes of the copper phase with FCC structure. Figure 11b presents the diffraction peak for the XRD patterns around 74°. Table 2 lists the 2θ corresponding to the different peak positions. The diffraction peak of the remelting specimen moved toward the low diffraction angle. The lattice constants for the remelting and non-remelting process specimens calculated by XRD were 3.61989 and 3.61753 Å, respectively. The lattice constant of pure copper is 3.6147 Å.

### 3.3. Characterization of Mechanical, Electrical, and Thermal Properties

Table 3 displays the hardness test results for the remelting and non-remelting specimens. The average hardness for both specimens was 92 HV_0_._3_, demonstrating that various processes have an insignificant effect on average hardness. However, the hardness measurement standard deviation under the remelting process was 8 HV_0_._3_, with a maximum value of 106 HV_0_._3_ and a minimum value of 80 HV_0_._3_, while under the non-remelting process, it was 17 HV_0_._3_, with a maximum value of 116 HV_0_._3_ and a minimum value of 58 HV_0_._3_.

**Figure 12:**
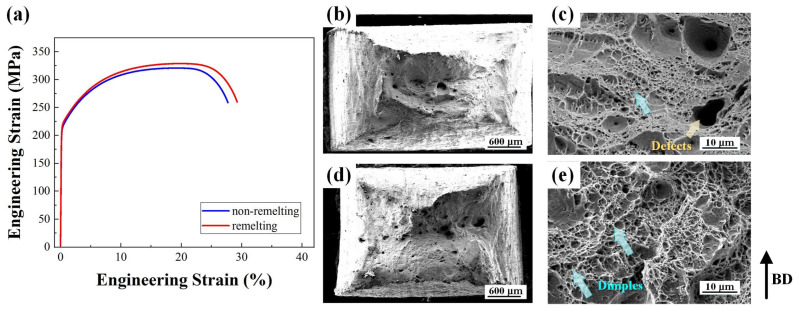
(**a**) Engineering stress–strain curves of different specimens, (**b**–**e**) SEM fractography of different specimens: (**b**,**c**) non-remelting and (**d**,**e**) remelting process specimens.

Figure 12 presents the tensile properties and fracture morphology of the non-remelting and remelting processes. For the remelting specimen, the yield strength (YS) was 222 MPa, while it was 214 MPa for the non-remelting specimen. The ultimate tensile strength (UTS) of the remelting specimen reached 329 MPa. The final elongation (EL) of the specimens reached 32%. Some defects were present on the fracture of the non-remelting process, as shown in Figure 12c. Dimples are uniformly distributed on the fracture in Figure 12e. Additionally, smaller dimples are scattered around larger ones, exhibiting ductile fracture characteristics.

In addition to the mechanical properties, Table 4 illustrates the electrical and thermal conductivities of the different specimens. The remelting process had better electrical and thermal conductivities than the non-remelting process.

## 4. Discussion

### 4.1. MPL Model and Parameter Optimization

Dimensional measurements were performed to create the MPL model. Combined with the analysis of the OM diagram (Figure 4), when the lap rate of the molten pool was less than 3 (R_L_ < 3), incomplete fusion defects were more likely to develop. Conversely, when the lap rate of the molten pool was greater than 9 (R_L_ > 9), the keyhole defect was more likely to occur. This was due to the heat input throughout the manufacturing process. Lap conditions, energy density, and subsequent heat accumulation have an impact on defects [17]. Excessive heat input increases the depth of the molten pool and vaporizes the elements, which stops the pores in the molten pool from overflowing in time. Furthermore, the imbalance between the molten pool sizes and their interval spaces is mostly responsible for incomplete fusion defects. Particularly, continuous incomplete fusion defects form when the heat input is continually insufficient, as shown in Figure 4d.

The corresponding molten pool sizes of different parameters were fitted to obtain adequate model data. The model was further employed to obtain more molten pool lap cases. Based on the relationship between R_L_ and defects, defect-free lap cases and their corresponding molten pool sizes were obtained. Thereafter, optimal process parameters were determined. Previous studies have shown that laser power, hatching space, and scanning speed affect the density, and thus, the study of a single variable is not accurate [29,30]. Most studies have attributed the factors to the volume-based energy density E [31,32,33,34,35]. Similar to other studies, LOF defects occurred when E was insufficient, and keyhole defects appeared when E was too high. The remelting process provides a wide adjustable range that presents a better process window than that obtained by just adjusting the laser energy density [17,23]. According to the optimal range of R_L_, the remelting process broadened the range of the preferred process parameters. As shown in Figure 3b, the concentrated optimal process window suggests that the parameter combination used in the remelting process is more likely to achieve high densities.

**Figure 13:**
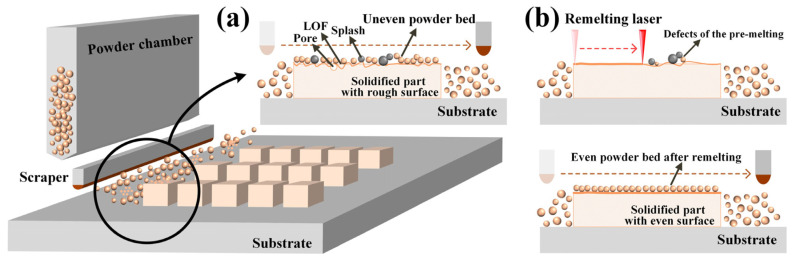
Schematic of the powder bed coating surface: (**a**) uneven surface caused by defects and (**b**) even powder bed coating after remelting.

This possibility mainly stemmed from the high laser reflectivity of CuCrZr powder and the high thermal conductivity of the alloy. During the non-remelting process, the formed surface was especially rough due to the splash that stuck to the face of the solidified molten pool. Simultaneously, the large splash impaired the powder fusion of the next layer, yielding an uneven powder bed surface, as shown in Figure 13a.

The defects can be resolved through the remelting process. That is, even if a defect (LOF, pore, or splash) is present during the pre-melting process, the subsequent remelting process will remelt the surface and make it uniform. As shown in Figure 13b, the splash particles were melted into the molten pool, and the pores overflowed during the remelting process to yield a uniform and smooth surface. Moreover, the laser of the remelting process acted on the surface that solidified after pre-melting rather than directly acting on the loose powder. The molten pool size was smaller during the remelting process than that during the pre-melting process due to low laser absorption, which preserved the regularity of layer overlapping without alteration.

### 4.2. Microstructural Evolution and Internal Mechanisms

Owing to the layer-by-layer melting characteristics, heat transfer frequently tended to follow the building direction, which explains why the grains extended upward from the dense part and grew into columnar crystals. Figure 7 shows that the remelting process affords smaller grains than the non-remelting process, despite all the grains being columnar crystals. In other words, the remelting process refines the grains. The remelting process plays a decisive role in the formation of fine grains, fine precipitates, and defects and has the potential to enhance the density. This is caused by the convection of fluids in the molten pool induced by the remelting process, the increased thermal conductivity due to the difference in the interaction of the laser with the powder and metal, and, most importantly, the faster cooling rate of the remelting process [36,37,38,39,40,41,42]. The specific kind of coagulated cells were formed due to the rapid coagulation rate of the PBF-LB/M process, which has also been reported in other relevant studies [43].

**Figure 14:**
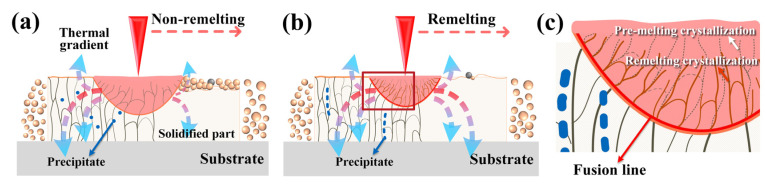
Schematic of molten pool behavior: (**a**) non-remelting process, (**b**) remelting process, and (**c**) the enlarged view of the molten pool of (**b**).

Thermal gradient is an important cause of grain refinement. Remelting has a substantial impact on the prior layer’s thermal history. The high thermal conductivity of the CuCrZr alloy caused the temperature of the previous melt track to drop too much, resulting in an insufficient energy input in the subsequent melt track [11]. The remelting process has twice as many thermal cycles as the non-remelting process, with a fixed construction height and layer thickness. Moreover, compared to the non-remelting process, during the remelting process, the heat input in a single pass is smaller, the molten pool size is smaller, and the heat transfer of solids is more effective than that of loose powders, which leads to a faster cooling rate in the solidification process and exacerbates the thermal gradient, as displayed in Figure 14a,b. Numerous dislocations occur during solidification due to the typical temperature field for nonlinear transient heat conduction, convection, and radiation during PBF-LB/M manufacturing. As shown in Figure 7f, the remelting process yields a higher GND density (1.12 × 10^14^) than the non-remelting process.

The remelting process eliminated the potential for a few of the columnar crystals to extend and grow. As displayed in Figure 14c, remelting caused convection in the fluid in the molten pool, forcing the previously formed columnar crystals to remelt and peel off into the new molten pool, which caused the molten pool to recrystallize. The solidified structure competed for growth space, and the main crystallization direction close to the temperature gradient became dominant. The fast cooling rate of the remelting process provided more opportunities for grain growth during solidification and, therefore, led to grain refinement.

Moreover, the remelting process afforded more thermal deformations. The stress and strain produced by thermal deformation dictated the density of dislocation. Dislocation cells, as shown in Figure 8d,h, were formed due to the high density of dislocations (Figure 7) and unbalanced thermomechanical conditions during solidification. Typically, dislocation windings form dislocation walls, which continue to form dislocation cell structures as residual stress develops due to solidification and heat shrinkage.

### 4.3. Performance Improvement Mechanism

In this study, the remelting process yielded finer grains (126.1–85.6 μm), smaller nanoprecipitates (107–77 nm), denser dislocation windings, and smaller dislocation cells (1200–633 nm) compared to the remelting process of previous studies, which is the main reason for its better performance. With a final elongation (EL) of 32%, the ductility significantly improved compared to that of the wrought CuCrZr counterparts (15%) [44]. The defects on the fracture of the non-remelting process stemmed from the low energy density during the PBF-LB/M manufacturing process. During PBF-LB/M processes, the energy density typically controls the effectiveness and speed of melting and bonding of the metal powders. When the energy density is very low, powders cannot be completely melted and bonded, resulting in fractures, porosity, and other faults in the component, which affects their performance and quality. The yield strength (YS) equation can be used to calculate the influence of various influencing mechanisms on the mechanical properties of the specimen [11,21,45,46,47]:

(4) $$YS=\sigma_{0}+∆\sigma_{gs}+∆\sigma_{ps}+∆\sigma_{ds}+∆\sigma_{ss}$$

where *σ*_0_ is the Peierls–Nabarro stress or the friction lattice for pure copper (20 MPa), Δ*σ_gs_* is the contribution of the grain refinement, Δ*σ_ps_* is the precipitation strengthening, Δ*σ_ds_* is the dislocation strengthening, and Δ*σ_ss_* is the solid solution strengthening. The strengthening mechanisms include fine-grained, precipitation, dislocation, and solid solution strengthening. Figure 7f and Figure 8 demonstrate that the PBF-LB/M process increases dislocation density, leading to dislocation walls and dislocation cells. Smaller dislocation cells provide more guarantee for the high performance of the as-built alloy. They can be regarded as dense walls of dislocation density, and dislocation slipping may be inhibited by these cellular microstructures during deformation [48,49]. In other words, compared to previous studies, the control of the manufacturing process in remelting enabled the variation of the dislocation structure and enhancement of the mechanical properties. However, the tensile properties of the remelting process were not significantly improved compared to those of the non-remelting process. This is due to the difference in the precipitated phases. The precipitated phases of the non-remelting process were Cr_2_Zr and Cu_5_Zr, while the precipitated phase of the remelting process was only Cu_5_Zr, as shown in Figure 10e. This is consistent with the solute content results from XRD. Thus, there are fewer precipitation strengthening effects during the remelting process. This also explains why the remelting specimen’s average hardness does not increase.

As is well known, porosity and impurities significantly affect conductivity [11]. In this study, the conductivity of the non-remelting specimen was 78% IACS, and the conductivity of the remelting specimen reached 96% IACS, which is higher than that obtained by other studies on the Cu alloy [13,50,51,52]. The thermal conductivity calculated by Equation (3) was also higher than that obtained by other studies. Mattiessen’s rule can be used to express the resistivity at room temperature and calculate conductivity:

(5) $$r=r^{\prime }+∆\sigma_{GR}+∆\sigma_{ps}+∆\sigma_{ss}+∆\sigma_{ds}$$

where *r* represents various resistivities stemming from electron scattering on grain boundaries, precipitates, solid solutions, and dislocations [21,45].

The evolution of metal conductivity can be classified into three theories: classical free electron theory, quantum electronics theory, and solid band theory. During the remelting process, the alloy quickly solidifies, as discussed in Section 4.2. Increasing the cooling rate can improve the solubility of alloying elements in the matrix, leading to the formation of a supersaturated solid solution [53]. The XRD results verify this. All diffraction peaks of the remelting specimen moved toward the low diffraction angle. This can be explained by Bragg’s law [Equation (6)], where *d_hkl_* is the lattice spacing, *θ* is the diffraction angle, *n* is a positive integer, and *λ* is the wavelength of the XRD beam.

(6) $$2d_{hkl}sin\theta =n\lambda =const$$

Equation (6) shows that when the lattice spacing *d_hkl_* increases, the angle *θ* decreases, and vice versa. Therefore, the remelting specimen had a higher lattice spacing than the non-remelting specimen. Solute atoms are responsible for this phenomenon. The high cooling rate during the remelting process increased the solubility of Cr atoms, resulting in more serious lattice deformation. There are several factors that can affect the conductivity of metals, including electrons and lattices [54]. Some studies have shown that an increase in solid solubility leads to severe matrix lattice distortion, which reduces the mean free path of electrons, resulting in a decrease in conductivity [55]. However, this study contradicts this conclusion, which might be explained by the slight variation in solute content. For alloys of the same system in this study, the effect of lattice distortion caused by different solid solution element contents is negligible. Furthermore, the average size of the precipitates in the non-remelting specimen was 107 nm, and in the remelting specimen was 77 nm. The mean free path of electrons in copper alloys is 39 nm [56]. According to Equation (5), the precipitate size affects electron scattering. The precipitation of Cu_5_Zr contributes to the increase in conductivity [57]. Thus, the conductivity of the remelting specimen was higher than that of the non-remelting specimen.

This study shows better mechanical properties, especially electrical conductivity. The remelting strategy represents a useful choice for components with high conductivity properties. However, this study needs to be further deepened because the improvement in mechanical properties in this study is not as pronounced as the increase in electrical conductivity. The remelting process is more time-consuming and costly. If electrical conductivity and mechanical properties can be improved at the same time, it will make up for its shortcomings. Therefore, we will continue to carry out process exploration that is conducive to performance improvement in the future. 

## 5. Conclusions

In this study, a CuCrZr alloy with high mechanical and electrical properties was obtained through L-BPF technology under the remelting process, which provided a solution for laser AM of high laser reflectivity powders and solved the problems of poor forming quality in copper alloy powders. The main conclusions are as follows:

(1) The optimal parameters for the process window were obtained using the MPL model to count the R_L_ of the molten pool and combine it with the microstructure. The MPL model provided scientific and efficient theoretical assistance for process optimization. The optimal process parameters for remelting were as follows: *P* = 330 W, *v* = 400 mm/s, and *h* = 0.09 mm.

(2) The remelting process generated an extremely conductive CuCrZr alloy that had a higher relative density and more uniform hardness than the non-remelting specimens. Notably, its electrical conductivity reached 96%, which is higher than that obtained by previous studies. Simultaneously, the remelting process yielded good mechanical properties: HV_0_._3_ of 92 HV_0.3_, UTS of 329 MPa, YS of 222 MPa, and an EL of 32%.

(3) The CuCrZr alloy microstructure was columnar, and the grains comprised a subcrystalline structure with a large number of dislocations. These dislocations were entangled, forming dislocation cells. Compared to the non-remelting process, the remelting process produced smaller grain sizes, smaller precipitates, denser dislocation lines, and smaller dislocation cells, and improved the solid solubility of Cr, so that there was no Cr phase precipitate, which enhanced electrical conductivity and mechanical properties.

## Figures and Tables

**Table 1:** Parameters for the process optimization window.

Process Parameters	Interval and Value	Remarks Description
*P* (W)	310, 330, 350, 370, 390	*t* = 30 μm
*v* (mm/s)	300, 400, 500	*d* = 60 μm
*h* (μm)	50, 70, 90, 70 *^re^*, 90 *^re^*	*re*: remelting

**Table 2:** XRD test results.

Manufacturing Process	Lattice Constant (Å)	Diffraction Angle 2θ (°)				
		Cu(111)	Cu(200)	Cu(220)	Cu(311)	Cu(222)
remelting	3.61989	43.254	50.375	74.007	89.780	94.975
non-remelting	3.61753	43.284	50.410	74.063	89.854	95.056

**Table 3:** Hardness measurements.

	A1	A2	A3	A4	A5		A1^re^	A2 ^re^	A3 ^re^	A4 ^re^	A5 ^re^	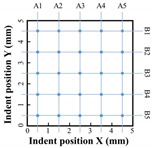
B1	93	94	89	105	105	B1 ^re^	92	99	89	84	102	
B2	82	73	96	109	116	B2 ^re^	103	97	89	81	99	
B3	58	64	79	94	108	B3 ^re^	99	104	82	85	80	
B4	61	103	116	106	102	B4 ^re^	87	85	93	81	96	
B5	66	76	101	114	98	B5 ^re^	91	93	83	99	106	
	AVG = 92 HV_0.3_ σ = 17 HV_0.3_						AVG = 92 HV_0.3_ σ = 8 HV_0.3_					

^re^: remelting.

**Table 4:** Summary of mechanical, electrical, and thermal properties.

Energy Density (J/mm^3^)	UTS (MPa)	YS (MPa)	EL (%)	Electrical Conductivity (%IACS)	Thermal Conductivity (W/mK)
524	326.5 ± 6	214 ± 5	33 ± 2	78 ± 2	324.5 ± 8
305 *^re^*	328.9 ± 8	222 ± 6	32 ± 2	96 ± 2	399.4 ± 4

*^re^*: remelting.

## Data Availability

Data available on request due to privacy/ethical restrictions.
